# Validating and assessing the oral health-related quality of life among Hungarian children with cleft lip and palate using Child-OIDP scale

**DOI:** 10.1007/s40368-020-00525-x

**Published:** 2020-04-22

**Authors:** S. Karki, J. Horváth, M.-L. Laitala, A. Vástyán, Á. Nagy, G. K. Sándor, V. Anttonen

**Affiliations:** 1 Research Unit of Oral Health Sciences, University of Oulu, Aapistie 3, PO Box 5281, 90220 Oulu, Finland; 2 Department of Dentistry, University of Pécs, Pécs, Hungary; 3 Department of Paediatrics, University of Pécs, Pécs, Hungary; 4 Medical Research Centre Oulu, Oulu University Hospital and University of Oulu, Oulu, Finland

**Keywords:** Cleft lip, Cleft palate, Hungary, Oral health, Quality of life

## Abstract

**Aims:**

To validate the Child-Oral Impact on Daily Performance (Child-OIDP) in the Hungarian language and to explore the oral health-related quality of life (OHRQoL) and associated factors among Hungarian children with cleft lip or/and palate (CLP).

**Methods:**

This cross-sectional study consists of a survey and clinical examination among conveniently selected children with CLP visiting the Pécs cleft lip and palate clinic, Pécs, Hungary. OHRQoL was assessed using the Hungarian version of Child-OIDP. Additionally, a validated structural questionnaire was used for gathering information related to oral hygiene practice. Clinical examination was done to register the dental status using ICDAS criteria, consequences of untreated dental caries (pufa), and bleeding on probing. Results were presented as proportions, means, and standard deviations (SD). Construct validity and internal reliability of the Hungarian Child-OIDP was assessed using the Pearson and Spearman's correlation coefficients, respectively. The logistic regression model examined the association between OHRQoL and explanatory variables.

**Results:**

A total of 45 children with CLP participated in this study. The Hungarian Child-OIDP had the Cronbach's alpha value 0.73, and the Spearman’s correlation coefficient was 0.31. The mean (SD) Child-OIDP score among the study participants was 4.4 (7.0) and three-fifths (65.9%, *n* = 27) of the participants reported impact in one or more items of the OHRQoL scale. Tooth brushing was more frequent among 6−10-year-olds compared to 11−16-year-olds. The proportion of those requiring restorative treatment need (DS ≥ 1) was 90.2% (*n* = 37), those with PUFA/pufa (score ≥ 1) was 24.4% (*n* = 10), and those with bleeding on probing (> 15%) was 63.4% (*n* = 26). Children aged 11–16 years had a higher impact on OHRQoL compared to the younger ones. Girls had a higher impact on OHRQoL compared to boys. Children with clefts involving both lip and palate had poorer OHRQoL than the rest. The same was true for those having a high dental caries rate.

**Conclusion:**

The Hungarian Child-OIDP was a reliable and valid measure. There was a substantial impact on OHRQoL among Hungarian children and adolescents with CLP. Age, gender, cleft type and dental caries were associated with poor OHRQoL.

## Introduction

Oral health-related quality of life (OHRQoL) is a subjective oral health indicator. These measures are commonly used for evaluating the functional, emotional, and psychosocial impact of oral diseases and disorders (Locker and Allen 2007). Children and adolescents with cleft lip with or without cleft palate (CLP) are more concerned with aesthetics and speech, therefore, having an impact on their quality of life (Queiroz Herkrath et al. 2015; Thomson and Broder 2018). Previous studies reported poor OHRQoL among children with CLP compared to their counterparts without clefts (Ward et al. 2013; Kortelainen et al. 2016) or compared to children with dental caries or orthodontic disorders (Jokovic et al. 2002; Khoun et al. 2018).

Globally, the incidence of the orofacial clefts was 195.5 thousand in 2017 (James 2018). During 2002–2006, the prevalence of CLP was 0.8 per 1000 live births worldwide, and 0.7 per 1000 live births in Hungary (Tanaka et al. 2012). A study conducted in Budapest, Hungary reported a high prevalence of dental caries, gingival bleeding, and congenitally missing teeth among children with CLP compared to their peers without CLP (Budai et al. 2001). The same was reported in another study conducted in Leipzig, Germany (Kirchberg et al. 2004). Moreover, these children are at risk of developing dental problems such as dental caries, and gingivitis (Wells 2013; Howe et al. 2015).

The Child-Oral Impact on Daily Performance (Child-OIDP) scale investigates the frequency and severity of oral impacts experienced in the past 3 months using a 3‑point Likert scale (Gherunpong et al. 2004). The eight psychometric properties (eating, speaking clearly, cleaning the mouth, sleeping, emotional stability, smiling, carrying out schoolwork, and contact with people) used in the Child-OIDP are considered to be brief, simple to understand, and easy to implement (Gherunpong et al. 2004). This scale has been previously validated in different cross-sectional settings (Zaror et al. 2019). However, a recent systematic review did not quote the availability of Child-OIPD in the Hungarian language (Zaror et al. 2019). Similarly, Gilchrist et al. (2014) in their systematic review concluded that one study in Hungary had used the Child-OIDP scale previously but the validation of Child-OIDP index in that study was questionable. Therefore, validation of Child-OIDP in the Hungarian language can be worthwhile. Additionally, only one study validated the Oral Health Impact Profile (OHIP-49) to evaluate the OHRQoL among adults (Szentpétery et al. 2006). To our knowledge, studies assessing OHRQoL among Hungarian children with CLP are nonexistent.

This paper aimed to validate the Child-OIDP in the Hungarian language and to evaluate the oral health-related quality of life among children with CLP using a validated Child-OIDP in the Hungarian language. Other aims were to measure the dental and gingival status and to explore their association with OHRQoL.

## Methods

### Study population

Children with CLP visiting the Pécs cleft clinic, Pécs, Hungary participated in this study. The cleft team in Pécs comprised two pediatric surgeons, a maxillofacial surgeon, orthodontists, speech therapists, and nurses. In the Pécs cleft clinic children under 17 years are treated. Children greater than 6 years of age who were accompanied by their parents/caretakers were included in this study. A total number of 44 children and parents were invited, of which 41 children participated in the study (Response rate 95%). This study was carried out from September 2017 to December 2018 during the follow-up visits of children with CLP.

### Oral health-related quality of life

Oral health-related quality of life was assessed using the Hungarian version of Child-OIDP. For the entire translation and validation process, the forward–backwards translation method was used. The study protocol was similar to a recent study in Nepal (Karki et al. 2018). First, two independent translators translated the original (English) version of the Child-OIDP into the Hungarian language, separately. Then, a draft was generated by compiling both versions by one of the members of the research team, which was later back-translated into English. Minor discrepancies in wording were revised after back-translation and all the members of the research group then verified the translated English versions. This consensus version was again translated into the Hungarian language and was considered as the final version.

Children were interviewed by a trained dentist (JH) for assessing the OHRQoL using the Hungarian Child-OIDP measure. For children below 10-years, parents or caretakers could assist in the interview. Initially, children were asked about the presence/absence of oral problems in the past 3 months, including the deformity of mouth or face. If the response was ‘no’, then no further interview was conducted, and all of the score was considered as 0. If the response was ‘yes’, then they were further asked about the severity and frequency for each daily performance (eating, speaking, cleaning the mouth, sleeping, maintaining emotional status, smiling, studying, and social contacts). The severity and frequency were scored on a 3-point Likert scale as 0 = no impact, 1 = little effect, 2 = moderate effect, and 3 = severe effect, and the frequency scores were 0 = no impact, 1 = once or twice a month, 2 = three or more times a month, or once or twice a week, 3 = three or more times a week. The impact score for each performance was calculated by multiplying the severity and frequency scores (range 0–9). The overall impact score was the sum of the impact scores of all eight performances (range 0–72). This overall score was divided by 72 and multiplied by 100 to obtain the total percentage score.

### Questionnaire

A structured questionnaire based on the World Health Organization (WHO) Oral Health questionnaire for children (WHO 2013) was used for collecting information related to oral hygiene practice. This questionnaire was translated in Hungarian language using the forward–backwards translation method. The information on tooth brushing frequency (twice daily/once daily/occasionally), use of toothbrush (yes/no), use of dental floss (yes/no), and use of fluoridated toothpaste (yes/no/don’t know) were included in the questionnaire. In addition, background information on age and gender were registered. All participants were interviewed for the gathering of information related to oral hygiene practice (*n* = 41).

### Oral examination

The clinical oral examination was done by two trained and calibrated dentists (SK, JH) using a dental mirror, and the WHO probe. The entire clinical oral examination was done in a clinical setting with a modern dental unit with light. No professional cleaning was done before the clinical examination, and the 3-in-1 syringe was used to blow-dry the teeth. One member was assigned for recording the oral findings in the datasheet.

### Training and calibration

Theoretical lectures on dental indices to be used for the oral examination were given to the clinical examiner before the calibration at the University of Oulu, Finland. The clinical examiners thereafter examined the 10 extracted teeth with varieties of carious lesions using the ICDAS criteria (Ismail et al. 2007). Finally, the clinical examination was done among four dental students and a child with cleft lip and palate at the University of Oulu and Oulu University Hospital, respectively. The inter-examiner agreement for dental caries detection ranged between fair (0.21) to excellent (0.84) agreements. Similarly, the intra-examiner agreement was calculated by examining the 10 extracted teeth ranged between 0.50 and 0.73. A reading manual with pictures illustrating the varieties of carious lesion was provided to the clinical examiners, however, no further reliability tests were conducted.

### Cleft types

The cleft groups were defined as suggested by Lithovius et al. (2014) as follows: hard palate cleft, soft palate cleft, right side lip and palate cleft, left side lip and palate cleft, bilateral lip and palate cleft, lip and alveolar cleft, and submucosal cleft.

### Dental status

Dental status measured the presence of carious lesions per surface using visual-tactile examination without radiographs. The International Caries Detection and Assessment System (ICDAS) was used for detecting caries lesions (Ismail et al. 2007). Each sound surface was considered as ICDAS code 0. The first visual changes in the enamel after drying was considered as ICDAS code 1 and the distinct visual changes in the enamel without drying was considered ICDAS code 2. The presence of signs of localized enamel breakdown due to caries with no visible dentin or underlying shadow was considered as ICDAS code 3, and those with underlying shadow indicating the progression of caries into dentin with or without localized enamel breakdown was considered as ICDAS code 4. Similarly, a distinct cavity with visible dentin was considered as ICDAS code 5, and extensive distinct cavity with visible dentin was considered as ICDAS code 6 (Ismail et al. 2007).

### Consequences of untreated dental caries

Consequences of untreated dental caries were registered using the PUFA/pufa index develop by Monse and colleagues (Monse et al. 2010). The index measured the visible pulp or pulp involvement (p/P), and/or ulceration of the oral mucosa as a result of root fragments (u/U), and/or a fistula (f/F), and/or an abscess (a/A) due to untreated dental caries. A per-tooth score was registered as well as the sum score which was calculated.

### Bleeding on probing (BOP)

The gingiva was examined for bleeding on probing using the WHO probes at six sites (distobuccal, mid buccal, mesiobuccal, distolingual, midlingual and mesiolingual). A per-tooth score was registered based on the presence or absence of gingival bleeding (WHO 2013).

### Ethical consideration

All the clinical examinations were conducted according to guidelines of the World Medical Association Declaration of Helsinki. The Medical University of Pécs gave ethical approval for the study (Permission no. 6924). Participation was voluntary and written consent was obtained from the parents/caretakers of the children.

### Statistical analysis

All data recorded on data sheets were transferred into an electronic data file for analyses. Data were analyzed using SPSS software (IBM SPSS Statistics for Windows, version 24.0. Armonk, NY: IBM Corp.), and RStudio^®^ version 1.1.383 (RStudio, Inc. Boston, MA).

Descriptive statistics were presented as proportions, means and standard deviations (SD). The ICDAS scores above score 3 with positive carious activity were considered as decayed surface (DS ≥ 1). Similarly, pufa/PUFA score 1 or more was considered as the presence of infection while bleeding on probing was categorized based on the presence of gingival bleeding in ≤ 15% or > 15% of teeth. The *χ*^2^ test was performed to compare the proportion between cleft groups, age and gender.

For the validity and reliability assessment, participants (*n* = 27) who had experienced oral problems in the past 3 months were only included in the analyses. To assess the reliability of the Hungarian Child-OIDP, Cronbach’s alpha was used as a measure for internal consistency and inter-item correlations were assessed using the Pearson correlation coefficient. For construct validity of Hungarian Child-OIDP, the association between Child-OIDP score and clinical dental status (absence or presence of decayed surface) was evaluated by the Spearman's correlation coefficient.

The overall Child-OIDP score was dichotomized as ‘no impact' score 0 and ‘impact' score ≥ 1. Logistic regression model examined the association between OHRQoL as an outcome variable, and age group (6−10 years vs 11−16 years), gender (boy vs girl), cleft type (cleft palate vs cleft lip and palate), dental status (total decayed surface), consequences of untreated dental caries (pufa + PUFA score), and bleeding on probing as explanatory variables. Both unadjusted and adjusted odds ratio (OR) and 95% confidence interval (95% CI) were calculated. For all analyses, *p* < 0.05 was considered statistically significant.

## Results

Age of the participants (*n* = 41) of this study ranged from 6- to 16-year-olds with mean (SD) age 11.3 (3.0). Most of the participants were boys (68.3%, *n* = 28) and had left side cleft palate involving lip (31.7%, *n* = 13) followed by bilateral cleft lip and palate (26.8%, *n* = 11). No difference in prevalence of dental caries was discovered between the cleft types. However, the prevalence of dental caries varied from 8 to 35% (Table 1).

**Table 1:** Distribution (%) of cleft types among the study participants stratified with gender and decayed surface

Cleft types	Gender		*p *value	Decayed surface		*p *value
	Boys (*n* = 28)	Girls (*n* = 13)		0 (*n* = 4)	≥ 1 (*n* = 37)	
Lip and alveolus (*n* = 5)	10.7	15.4	*0.610*	25.0	10.8	*0.082*
Hard palate (*n* = 5)	10.7	15.4		50.0	8.1	
Lip and palate (left) (*n* = 13)	28.6	38.5		–	35.1	
Lip and palate (right) (*n* = 3)	10.7	–		–	8.1	
Lip and palate (bilateral) (*n* = 11)	32.1	15.4		–	29.7	
Lip (*n* = 4)	7.1	15.4		25.0	8.1	
Total (*n* = 41)	68.3	31.7		9.8	90.2	

Italic values indicate *p* < 0.05

The inter-item correlation coefficients ranged from 0.01 (between speaking impact and contact/interaction impact) to 0.98 (between smiling and sleeping; and contact/interaction and sleeping) and the Standardized Cronbach’s alpha value was 0.73 (Tables 2, 3). The total Child-OIDP score was correlated with dental caries status (*ρ* = 0.313; *p *value = 0.046).

**Table 2:** Pearson correlation coefficients of psychometric properties of the Child-OIDP index among the study participants (*n* = 41)

Performance scores	Eating food	Speaking clearly	Cleaning mouth	Sleeping or relaxing	Maintaining usual emotional state	Smiling or laughing	Carrying out schoolwork	Contact with people
Eating food	1.00							
Speaking clearly	− 0.17	1.00						
Cleaning mouth	0.15	0.29	1.00					
Sleeping or relaxing	0.05	− 0.07	− 0.07	1.00				
Maintaining usual emotional stability	0.11	− 0.05	0.21	0.82**	1.00			
Smiling or laughing	0.12	− 0.09	− 0.08	0.98**	0.78**	1.00		
Carrying out schoolwork	–	–	–	–	–	–	1.00	
Contact with people	0.03	− 0.01	0.06	0.98**	0.83**	0.95**	–	1.00

**p < 0.001

**Table 3:** Internal reliability analysis: Cronbach’s alpha, Standardized alpha, corrected item—total correlation and alpha if item deleted

Items	Corrected item—total correlation	Alpha if item deleted
Eating food	0.04	0.70
Speaking clearly	− 0.05	0.74
Cleaning mouth	0.20	0.61
Sleeping or relaxing	0.67	0.48
Maintaining usual emotional state	0.67	0.46
Smiling or laughing	0.67	0.48
Carrying out schoolwork	0.00	0.61
Social contact	0.72	0.46
Cronbach’s alpha	0.61	
Standardized Alpha	0.73	

Two-thirds (65.9%, *n* = 27) of the participants reported having an impact on one or more items. Most of them reported an impact on eating (36.6%, *n* = 15) followed by speaking clearly (22%, *n* = 9) and maintaining their emotional state (22%, *n* = 9). The mean (SD) Child-OIDP score among the study participants was 4.4 (7.0). None of the participants had an impact on schoolwork (Table 4). The mean (SD) Child-OIDP score was high among oldest age group (11–16 years), among girls, those with cleft of lip and alveolus and left side lip and palate cleft, and those requiring restoration (Table 5).

**Table 4:** Distribution (%) and mean (SD) Child-OIDP scores and daily performances among study participants

	Eating food	Speaking clearly	Cleaning mouth	Sleeping or relaxing	Maintaining usual emotional stability	Smiling or laughing	Carrying out schoolwork	Contact with people	Overall impact
Prevalence (%)	36.6	22.0	19.5	2.4	22.0	4.8	–	4.8	65.9
Impact score									
Mean (SD)	1.2 (2.4)	1.2 (2.6)	0.6 (1.5)	0.2 (1.4)	0.6 (1.6)	0.3 (1.4)	–	0.3 (1.4)	4.4 (7.0)

**Table 5:** Mean (SD) and range Child-OIDP score stratified with age, gender, cleft type, decayed surface, pufa/PUFA score, and BOP

	Child-OIPD score	
	Mean (SD)	Range
Age		
6–10 years (*n* = 16)	2.6 (3.3)	11
11–16 years (*n* = 25)	5.5 (8.5)	38
Gender		
Boys (*n* = 28)	3.3 (4.0)	13
Girls (*n* = 13)	6.7 (11.0)	38
Cleft types		
Lip and alveolus (*n* = 5)	6.6 (6.7)	16
Hard palate (*n* = 5)	0.4 (0.6)	1
Lip and palate (left) (*n* = 13)	6.1 (11.0)	38
Lip and palate (right) (*n* = 3)	0.6 (1.1)	2
Lip and palate (bilateral) (*n* = 11)	4.0 (3.1)	10
Lip (*n* = 4)	4.8 (4.3)	8
Restorative treatment need		
No	0.3 (0.5)	1
Yes	4.8 (7.3)	38
Consequences of untreated dental caries		
pufa/PUFA = 0	4.6 (7.8)	38
pufa/PUFA ≥ 1	3.7 (4.0)	10
Bleeding on probing		
BOP ≤ 15%	5.7 (5.2)	16
BOP > 15%	3.6 (7.9)	38

Among the study participants, mean DS (SD) and mean pufa (SD) scores were 8.5 (10.6) and 0.6 (1.3), respectively. The proportion of those requiring restorative treatment (DS ≥ 1) was 90.2% (*n* = 37) and that with PUFA/pufa score ≥ 1 was 24.4% (*n* = 10). Both tooth decay and gingival bleeding were high among the children aged 11−16 years. Tooth brushing was more frequent among 6- to 10-year-olds compared to 11- to 16-year-olds. Two-thirds of the participants did not know if their toothpaste contained fluoride, of which most were boys and those belonging to 11- to 16-year-old age group. Missing teeth was the most common of the dental anomalies (70.7%, *n* = 29), followed by supernumerary teeth (22.0%, *n* = 9) (Table 6).

**Table 6:** Distribution (%) of decayed surface, pufa/PUFA score, BOP and oral hygiene behaviours among study participants stratified with age and gender

	Age		*p* value	Gender		*p* value	Total (*n* = 41)
	6–10 years	11–16 years		Boys	Girls		
Restorative treatment need							
ICDAS score ≥ 3	75.0	100.0	*0.008*	89.3	92.3	*0.762*	90.2
Consequences of untreated dental caries							
Pufa/PUFA ≥ 1	31.2	20.0	*0.413*	28.6	15.4	*0.360*	24.4
Bleeding on probing							
BOP > 15%	43.7	76.0	*0.036*	64.3	61.5	*0.865*	63.4
Tooth brushing frequency							
Twice daily	62.5	44.0	0.248	42.9	69.2	0.116	51.2
Use of toothbrush							
Yes	93.8	92.0	0.834	89.3	100.0		
Use of dental floss							
Yes	6.3	16.0	0.352	7.1	23.1	0.147	12.2
Use of fluoridated toothpaste							
Yes	43.8	24.0	0.185	21.4	53.8	0.038	31.7
Don’t know	56.3	76.0		78.6	21.4		68.3
Developmental malformation							
Missing teeth	68.8	72.0	0.823	75.0	61.5	0.378	70.7
Supernumerary teeth	18.8	24.0	0.692	10.7	46.2	0.011	22.0
Morphologic changes	12.5	16.0	0.757	17.9	16.7	0.391	14.6
Hypoplastic	12.5	24.0	0.365	14.3	30.8	0.215	19.5
Hypomineralization	12.5	8	0.636	7.1	15.4	0.408	9.8

Italic values indicate *p* < 0.05

Despite statistical non-significance, children aged 11−16 years had a high impact on OHRQoL compared to the younger ones (6−10 years). Girls had a higher impact on OHRQoL compared to boys. Children with clefts involving both lip and palate had Child-OIDP score ≥ 1, indicating poor OHRQoL. The same was true with those having a high rate of dental caries, even though statistically significant association was not evident (Table 7).

**Table 7:** Association between Child-OIDP score and socio-demographic characteristics, cleft type, dental status, consequences of untreated dental caries, bleeding on probing on OHRQoL

Explanatory variable	Child-OIDP score ≥ 1	
	UOR (95% CI)	AOR (95% CI)
Age		
6–10 years	1	1
11–16 years	1.26 (0.34–4.79)	1.31 (0.25–6.86)
Gender		
Boys	1	1
Girls	2.16 (0.52–11.24)	2.45 (0.51–11.82)
Cleft type		
Cleft palate	1	1
Cleft lip and palate	3.41 (0.50–23.36)	3.45 (0.43–27.60)
Restorative treatment need	1.02 (0.95–1.10)	1.04 (0.92–1.18)
Consequences of untreated dental caries	1.09 (0.63–2.22)	0.81 (0.29–2.27)
BOP	0.95 (0.83–1.08)	0.93 (0.79–1.07)

*OR* unadjusted odds ratios, *AOR* adjusted odds ratios, *95% CI* 95% confidence interval

## Discussion

This study indicates that the Hungarian version of the Child-OIDP was reliable and valid for investigating oral health-related quality of life (OHRQoL) among children with cleft lip and palate (CLP). Oral health has a considerable impact on quality of life among this group of patients. This may be due to the fact that in addition to the cleft they also commonly had dental caries and gingival bleeding, which was in line with a prior study (Budai et al. 2001).

Concerning the validation process, the forward–backwards translation method as suggested by Guillemin and colleagues (Guillemin et al. 1993) was followed here, which also ensured the face and content validity. A similar protocol was also followed for cross-cultural adaptation and validation of the original instrument (Gherunpong et al. 2004) and in other previous validation studies (Karki et al. 2018; Alzahrani et al. 2019). Both alpha values and correlation coefficients are within acceptable ranges that demonstrate the Hungarian Child-OIDP to be reliable (Nunnally and Bernstein 1994). However, caution is necessary when comparing studies with different populations. As none of the participants noted any impact on schoolwork, correlation coefficient value for this item was null. Deletion of eating and speaking items would have increased the alpha values. However, the authors did not consider it necessary to delete as most of the participant reported having problems with both eating and speaking. The overall pattern of the items Child-OIDP was somewhat different compared with children without CLP (Gherunpong et al. 2004; Karki et al. 2019). This is most likely due to different causes of impact on OHRQoL in these groups. Those with CLP have dental caries with its consequences; however, esthetic problems play a more important role among CLP children.

The mean Child-OIDP score among the current study is low compared to Thai children with CLP (Pisek et al. 2014). Pisek et al. (2014) included children with CLP aged 10- to 14-year-olds in their study. Similarly, a recent study from France found a high median Child-OIDP score among those with CLP (Friedlander et al. 2019). Friedlander et al. (2019) included children aged 6- to 17-year-olds with rare orofacial diseases. The Child-OIDP was developed for the 10- to 12-year-old Thai children (Gherunpong et al. 2004). Inclusion of younger children with proxy informants may lead to information bias as reported by Reissmann et al. (2017). Therefore, all the participants were interviewed as a primary informant in the study to minimize the occurrence of such potential bias.

Restorative treatment need and gingival bleeding were high among the present study population. A similar finding was reported in a previous Hungarian study conducted among children with CLP (Budai et al. 2001). Clinical examination in the present study followed ICDAS and BOP guidelines (Ismail et al. 2007; WHO 2013). These indices follow the same protocol irrespective of dentition. However, presenting the result for different dentitions combined in the present study is justified for having restorative treatment need as a co-variate despite the dentition. With a bigger study population, presenting results according to chronological age and dental stages would be valuable. Children with clefts involving both lip and palate were at risk of dental caries in this study. This finding is in line with a previous study from Northern Finland (Lehtonen et al. 2015). A recent systematic review also concluded that children with CLP have higher dental caries prevalence than their counterparts without CLP (Worth et al. 2017). The reason may be that the children born with a cleft are prone to dental caries and gingival diseases as they are at high-risk of poor oral hygiene (Wells 2013). Apart from that, the presence of dental anomalies may be another cause (Howe et al. 2015). Participants in this study had poor oral conditions. Almost all had dental caries and three-fourths had bleeding on probing. It can be speculated that in spite of their regular follow-up focus on oral health promotion was rare. High prevalence of dental caries and gingival bleeding in the older age group compared to the younger ones may be due to less frequent tooth brushing habits among them. In addition, most of the CLP patients undergo fixed orthodontic treatment that restricts oral hygiene thereby increasing dental and periodontal disease risk (Cheng et al. 2007). Concerning the prevalence of dental anomalies, our finding is similar to Lehtonen and colleagues. They also reported that missing teeth and supernumerary teeth were most prevalent dental anomalies among the Finnish children with CLP (Lehtonen et al. 2015).

The present study showed a high impact on OHRQoL among children with CLP. Similar findings were also reported previously (Ward et al. 2013; Kortelainen et al. 2016; Khoun et al. 2018). Impact on eating was the most reported item in this study followed by the speaking and maintaining the emotional stability. This is fully in line with a systematic review which reported that eating, speaking and emotional well-being are the most influenced dimensions of OHRQoL among children and adolescents with CLP (Queiroz Herkrath et al. 2015). The reason for the impact on eating may be restricted orofacial function among the study participants as suggested by Sundell and Marcusson (2019). High impact on speaking/speech among the study participant may be due to the presence of cleft palate that restricts speech. Children with CLP are prone to velopharyngeal dysfunction leading to speech problems (Woo 2012). Concerning the impact on maintaining the emotional stability in this study, scarred upper lip may be a cause. However, the emotional component is closely related to psychological issues that comprise self-esteem, appearance and family support (Kapp-Simon 2004). Furthermore, the emotional aspect of children with CLP reported having conflicts (anxiety and fear) toward their family compared to their counterpart children without CLP (Kasuya et al. 2000). To understand this aspect of quality of life may require further detailed and careful study.

One of the strengths of this study was assessing the OHRQoL using the validated Hungarian version of Child-OIDP to our knowledge for the first time. Another strength would be the inclusion of both subjective and clinical components of oral health together. However, the cross-sectional nature of this study is one of the limitations. Another limitation would be not conducting a pre-pilot study during the translation process. Likewise, not considering the sample size calculation and not identifying the protective factors for OHRQoL among this group would be other limitations. A control group would have added the value of this study. There was variation (low to excellent) in the kappa values representing inter-examiner agreement in this pilot study. In future studies, emphasis must be given to ensure high-quality training and calibration.

The findings from this study may highlight the crucial role of the person him/herself, the family as well as clinicians in maintaining good oral health thereby improving quality of life. Clinicians must keep in mind that these children encounter the cleft team repeatedly including dentists until their adulthood. It is most important to focus on preventive procedures, as these children are even more at risk of oral diseases than children without cleft (Worth et al. 2017). Future study should incorporate improvements in OHRQoL following each treatment including the speech therapy and psychological counselling. It should also include resilience or protective factors among children and adolescents with CLP.

## Conclusion

In conclusion, the Hungarian Child-OIDP was reliable and valid to measure the oral health-related quality of life. Hungarian children with orofacial clefts frequently have dental caries and gingivitis. There is a substantial impact on OHRQoL among children and adolescents with CLP. This is most specifically related to eating, speaking and maintaining emotions. Therefore, the role of the cleft team may be vital in terms of minimizing these impacts while giving these children all the possible help they need.
